# Trends in paediatric rheumatology referral times and disease activity indices over a ten-year period among children with juvenile idiopathic arthritis: results from the childhood arthritis prospective study

**DOI:** 10.1186/1546-0096-12-S1-P13

**Published:** 2014-09-17

**Authors:** Flora Mcerlane, Helen E Foster, Roberto Carrasco, Eileen Baildam, SE Alice Chieng, Joyce Davidson, Yiannis Ioannou, Lucy R Weddurburn, Wendy Thomson, Kimme L Hyrich

**Affiliations:** 1 Paediatric Rheumatology, Great North Children's Hospital, Newcastle upon Tyne, UK; 2 Arthritis Research UK Epidemiology Unit, University of Manchester, Manchester, UK; 3 Rheumatology, Institute of Cellular Medicine, Newcastle University, Newcastle upon Tyne, UK; 4 Paediatric Rheumatology, Alder Hey Children's Hospital, Liverpool, UK; 5 Paediatric Rheumatology, Royal Manchester Children's Hospital, Manchester, UK; 6 Paediatric Rheumatology, University of Glasgow, Glasgow, UK; 7 Rheumatology, University College London, UK; 8 Rheumatology Unit, UCL Institute of Child Health, London, UK

## Introduction

The medical management of children and adolescents with juvenile idiopathic arthritis (JIA) has advanced significantly over the past ten years. The UK BSPAR Standards of Care (2009) stipulate that children with JIA should see a paediatric rheumatology team (PRh) within ten weeks of symptom onset and within four weeks of referral. It is not known how often these standards are met or whether they have impacted on outcomes.

## Objectives

To describe trends in referral times, baseline disease severity, treatment times and one-year outcomes over a ten-year period among children with new-onset JIA.

## Methods

The Childhood Arthritis Prospective Study is a prospective inception cohort of children with new-onset inflammatory arthritis recruiting from seven UK PRh centres. This analysis included all children recruited between 2001 and 2011 with at least one year of follow-up. The cohort was divided into four groups by year of diagnosis and baseline referral times, disease pattern, disease activity indices, time to first definitive treatment and proportion achieving minimal disease activity (MDA) (Magni-Manzoni, 2008) were determined for each group. Values across the four groups were compared using linear and logistic regression, adjusting for PRh centre and disease pattern.

## Results

Table 1.

**Table 1:** 

	2001-2004	2005-2006	2007-2008	2009-2011	P value
N (1066)	285	265	306	210	--

Time between symptom onset and 1^st^ PRh, weeks	22.7 (11.9, 40.1)	23.5 (12.1, 52.7)	24.7 (12, 58.2)	23.1 (13.2, 50.1)	0.28
% seen within 10 weeks	20.5%	21%	20%	19%	0.66

Time from referral to 1^st^ PRh appointment, weeks	3.4 (1.2, 7.9)	4 (1.4, 7.3)	4.7 (1.4, 8)	4.3 (1.6, 8.7)	0.61
% seen within 4 weeks	58%	55%	49%	50%	0.02

AJC	2 (1, 4)	2 (1, 6)	2 (1, 5)	2 (1, 5)	0.82

JADAS	10.8 (7, 16.2)	12.1 (7.2, 20.2)	9.3 (4.4, 18.4)	10.4 (4, 10.6)	0.60

CHAQ	0.8 (0.1, 1.5)	0.9 (0.3, 1.5)	0.6 (0.1, 1.4)	0.6 (0.1, 1.3)	0.045

Oligoarticular pattern: Time (days) from 1^st^ PRh to 1^st^ IA steroid, median (IQR)	25.5 (9,65)	25 (7, 49)	19 (8, 48)	19 (9, 48)	0.04
% in MDA at 1 year	62	66	66	67	0.87

Time (days) from 1^st^ PRh to 1^st^ methotrexate, median (IQR)	27 (1, 79)	17 (1, 43)	5 (0, 17)	11 (0, 84)	0.03
% in MDA at 1 year	39	56	50	59	0.07

Time (days) from 1^st^ PRh to 1^st^ methotrexate, median (IQR)	37 (14, 78)	14 (9, 25)	15 (6, 24)	15 (13, 26)	0.07
% in MDA at 1 year	64	50	75	57	0.85

## Conclusion

Approximately half of children with new-onset JIA were not seen within four weeks of referral, with only 20% within ten weeks of symptom onset. The reasons for the former may be related to PRh service pressures, with the latter relating to both public and physician education. Although it is encouraging to see more rapid introduction of treatment, further research is necessary to understand why approximately one-third of children have active disease at one year. Delay in access to definitive treatments may impact on outcomes.

## Disclosure of interest

None declared.

